# Ocular inflammatory adverse events of immune checkpoint inhibitors- a multimodal imaging study

**DOI:** 10.1186/s12348-026-00627-5

**Published:** 2026-08-12

**Authors:** Srinivasan Sanjay, Loo Yunhua, Samanthila Waduthantri, Chee Soon-Phaik

**Affiliations:** 1 Department of Cataract and Comprehensive Ophthalmology, Singapore National Eye Centre, 11 Third Hospital Avenue, 168751 Singapore; 2 Department of Ocular Immunology and Inflammation, Singapore National Eye Centre, 11, Third Hospital Avenue, 168751 Singapore; 3 Singapore Eye Research Institute, The Academia, Level 6, Discovery Tower, 20 College Road, 169856 Singapore; 4 Department of Ophthalmology Yong Loo Lin School of Medicine, NUHS Tower Block, Level 7, National University of Singapore, 1E Kent Ridge Road, 119228 Singapore

**Keywords:** Immune checkpoint inhibitors, Uveitis, Ocular inflammation, Immune-related adverse events, Panuveitis, Pembrolizumab, Nivolumab, Ipilimumab

## Abstract

**Purpose:**

To describe the spectrum, clinical course, and outcomes of ocular inflammatory adverse events linked to immune checkpoint inhibitors (ICIs) in a Singapore cohort.

**Methods:**

This retrospective cohort examined six patients who developed ocular symptoms after ICI treatment, including ipilimumab-nivolumab and pembrolizumab. We analyzed clinical features, symptom onset timing, diagnoses, treatments, and outcomes.

**Results:**

Six Chinese Singaporean patients (2 men, 4 women; aged 52–72 years) presented with eye redness, pain, and blurred vision 2–14 days after ICI doses. Symptoms arose after the first dose in one patient and after the second to tenth doses in the others. Diagnoses included anterior uveitis (*n* = 3; two granulomatous) and granulomatous panuveitis (*n* = 3), with initial best-corrected visual acuity from 20/25 to 20/100. All received topical and/or systemic corticosteroids without needing to stop ICIs. One patient had anterior uveitis recurrence 3 months later, with both episodes resolving using topical steroids. Two panuveitis cases progressed to sunset-glow fundus; another showed bilateral choroidal granulomas on angiography without visible clinical signs. Final visual acuity ranged from 20/20 to 20/50, with no significant long-term morbidity over 8–72 months of follow-up.

**Conclusion:**

Ocular inflammatory adverse events from ICIs in this Singapore cohort showed a predominantly granulomatous spectrum with favorable corticosteroid responses, enabling ICI continuation. Multimodal imaging highlighted choroidal granulomas and sunset-glow fundus evolution. Early recognition and multidisciplinary care preserved vision across diverse Asian patients without oncologic compromise.

## Introduction

Immune checkpoint inhibitors (ICIs) targeting cytotoxic T-lymphocyte-associated antigen 4 (CTLA-4) and programmed death-1 (PD-1)/programmed death-ligand 1 (PD-L1) have transformed treatment for advanced malignancies by enhancing antitumor T-cell responses [1, 2]. Cytotoxic T-lymphocyte-associated protein 4 is a transmembrane receptor found mainly on regulatory T cells and memory T cells [3]. It competes with the co-stimulatory molecule CD28 for binding to B7-1 (CD80) and B7-2 (CD86) on antigen-presenting cells (APCs) [3]. Programmed Cell Death Protein 1 (PD-1) is a cell surface protein expressed on natural killer (NK) cells, APCs (B cells, macrophages, dendritic cells), and activated T cells where it functions as a negative regulator of immune responses [2]. Ipilimumab a neutralizing antibody targets CTLA-4, a receptor that acts on the initial priming and activation of T cells in lymph nodes [2]. The CTLA-4 ipilimumab (Yervoy) was the first ICI approved by the FDA in 2011. PD-1 inhibitors such as nivolumab and pembrolizumab block inhibitory signaling in activated T cells, thereby reinvigorating antitumor immunity, but they differ slightly in structure, binding behavior, dosing schedules, and approved indications [2]. 

Immune checkpoint inhibitors are known to cause a range of side effects related to immune activation, commonly called immune-related adverse events (irAEs) [2]. The incidence of irAEs ranges between 64 and 72%, with high-grade IRAEs affecting up to 18–24% of patients undergoing ICI therapy. [4] Though most irAEs appear within 3–6 months of ICI initiation, delayed responses may take up to a year to appear, posing a challenge to diagnosis [5–7]. 

Ocular side effects associated with ICI therapy are generally rare and occur alongside other systemic irAEs. Though their combined incidence is only around 1%–2.8%, early detection is necessary to prevent significant impacts on patient’s vision and quality of life [8, 9]. Ocular side effects from ICI are often underestimated in clinical trials, though they are reported in some phase III studies and numerous case reports [1]. The most common manifestations include uveitis, orbital inflammation, and ocular surface alterations. Early ophthalmic evaluation and timely corticosteroid treatment are critical to improving visual outcomes for these patients [1]. Most ocular irAEs were reported in patients undergoing treatment for advanced or metastatic melanoma [8]. Only one small phase I study for combined nivolumab and ipilimumab in advanced melanoma reported more than one case of high-grade uveitis (two cases, or 4%). [8, 10] Risks of uveitis are significantly higher in combined therapy than in monotherapy. In lung cancer patients, the risk of developing uveitis during their treatment course is more than seven times higher amongst those receiving combined programmed death ligand 1(PD-L1)/CTLA-4 inhibitors therapy than either ICI monotherapy [11]. 

Fortes et al. [9] in their cohort of 996 patients, ocular irAEs were seen in 2.8% of cases, with dry eye as the most common problem and uveitis the next most frequent. PD-L1–targeted drugs appeared to cause more ocular surface toxicity, but rates of uveitis and retinal complications were similar across checkpoint targets [9]. Importantly, most affected patients improved with treatment, and immunotherapy could usually be continued. In another major study of 962 Chinese patients Gan et al. [12], irAEs were common overall, but eye problems were uncommon, affecting just 1.1% during the first year. Lung cancer patients had a higher risk of both general and ophthalmic side effects, and ophthalmoplegia was the most frequent eye-related event in this cohort. Most cases responded well to steroid treatment, although stopping immunotherapy was followed by cancer progression in nearly half of the affected patients [12]. Alongside ophthalmoplegia, Vogt-Koyanagi-Harada (VKH)-like syndrome is increasingly reported in Asian demographics, presenting with bilateral granulomatous panuveitis that can eventually evolve into a classic “sunset glow” fundus [13]. The diagnosis and management of severe ocular irAEs—most notably VKH-like syndrome—have been greatly improved by the routine use of multimodal imaging. In the acute phase, patients frequently present with bilateral granulomatous choroiditis and, occasionally, secondary exudative retinal detachments [13]. Multimodal imaging is central in ICI-associated uveitis because it helps define the anatomic pattern and severity of inflammation beyond what slit-lamp examination alone can show [1, 2, 13]. Optical coherence tomography (OCT), fundus fluorescein angiography (FFA), indocyanine green angiography (ICGA), and fundus photography can reveal serous retinal detachment, choroidal thickening, optic disc leakage, and VKH-like changes that support the diagnosis [9, 13]. These modalities are especially useful when the presentation is subtle, atypical, or delayed, because they help distinguish immune-related disease from other inflammatory or masquerade syndromes [1, 2, 12]. Serial imaging also provides an objective way to monitor response to corticosteroids or treatment modification, especially when choroidal and subretinal findings improve over time [9, 12, 13]. B scan ultrasonography can detect sub retinal fluid and choroidal thickness in suspected VKH [13]. Overall, multimodal imaging improves diagnostic confidence and follow-up in this heterogeneous toxicity [1, 2].

Fortunately, topical and systemic corticosteroids remain highly effective for most of these ocular presentations [12, 13]. However, treating these severe inflammatory events often forces difficult decisions regarding the patient’s overall oncological care. To control the toxicities in the Chinese cohort, oncologists had to discontinue ICI therapy in most affected patients, which sadly resulted in cancer progression for nearly half of them [12]. 

Nivolumab-induced ocular inflammation is a rare but serious irAEs that requires ruling out other causes before management. Maintaining therapy is possible, provided there is close collaboration between oncologists and ophthalmologists to carefully weigh the risk-benefit ratio [14]. 

## Methods

This study was a retrospective cohort reviewing medical records of patients referred to our tertiary eye care centre for ocular inflammation following ICI therapy. We collected demographic data, cancer diagnoses, ICI regimens, symptom onset timing, clinical findings, investigations, treatments, and outcomes. Patients with anterior uveitis (Patients 1–3) presented within a week after onset of symptoms (ocular symptoms occurred 2–14 days after administration of ICI) at acute stage, while patients 4 and 5 were reviewed during their chronic stage. Both these patients had been treated earlier for bilateral panuveitis and their time of presentation following ocular symptoms was 8/10 days respectively (ocular symptoms occurred 14/10 days after administration of ICI respectively). Patient 6 had presented with blurring of vision in right eye more than the left and presented a day after onset of symptoms (ocular symptoms occurred 7 days after administration of ICI). Ocular inflammation was classified per Standardisation of Uveitis Nomenclature (SUN) criteria, noting anatomical location (anterior, intermediate, posterior, or panuveitis) and granulomatous (large mutton fat keratic precipitates) features. Best-corrected visual acuity (BCVA) was recorded at baseline and follow-up. Ultrawide field fundus photography (Optos™ Australia, South Australia, 5009) and OCT(Spectralis™ software V6 16.8 Heidelberg Engineering, Germany) was done for all patients. The fundus fluorescein angiography and indocyanine angiography (Optos™ Australia, South Australia, 5009) was done for 3 patients with panuveitis. All patients except patients 4 and 5 were seen at first presentation of symptoms. Patients 4 and 5 had been treated earlier with steroids when we first reviewed them.

All patients underwent standard uveitis workup, including full blood count, random blood glucose, erythrocyte sedimentation rate, and C-reactive protein, liver function and kidney function tests. Infective screening ruled out tuberculosis and syphilis. Pre-steroid evaluation included hepatitis B, hepatitis C, and HIV screening, chest X-ray, and urinalysis—particularly for panuveitis cases. During oral steroid therapy, we monitored blood pressure, body weight, and glucose levels. Management details—including topical versus systemic corticosteroids and ICI continuation—were documented. The cohort was included in an ethics board-approved study (Number 2019-2811-SSR3).

## Results

Six Chinese Singaporean patients (2 men and 4 women), aged 52–72 (median: 63.5) years, developed new-onset ocular inflammatory symptoms after treatment with ICIs. Presenting symptoms included ocular redness, pain, and blurred vision, with onset 2–14 days after ICI administration (median, 7 days). None of the patients had auditory or vestibular symptoms.

**Fig. 1:**
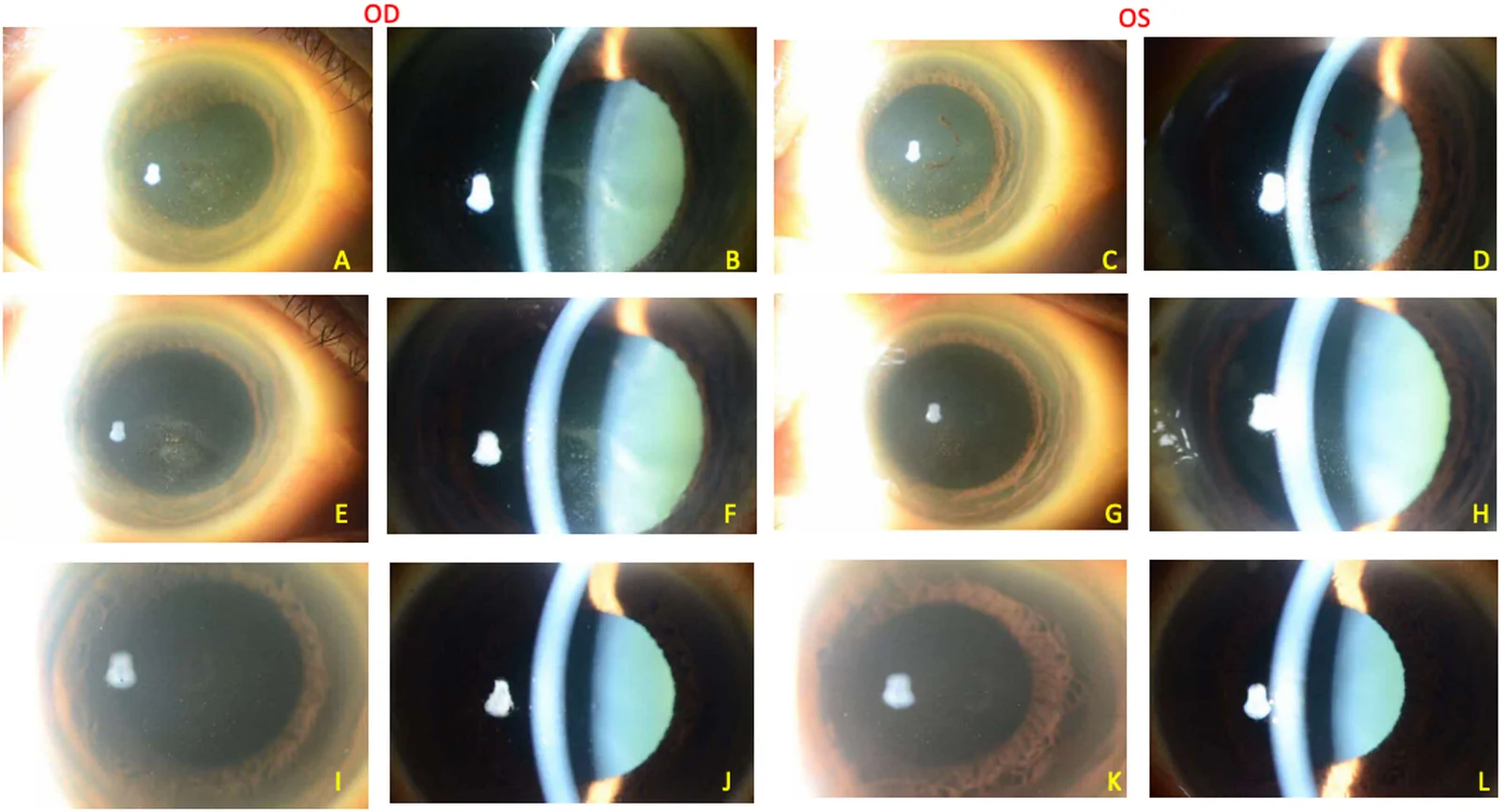
(A-H) shows granulomatous anterior uveitis in patient 2. **A**-**D** shows first presentation and **E**-**H** recurrent episode. **A**, **B** &**E**,**F** are images from right eye and **C**, **D** & **G**,**H** are from left eye. **I**-**L** shows non granulomatous anterior uveitis in patient 1

In one patient, symptoms began 14 days after the first dose; in the remaining patients, symptoms developed after the second to tenth doses, again within 2–14 days of treatment. The ICI regimens were ipilimumab-nivolumab combination therapy (*n* = 3) and pembrolizumab (*n* = 3). These agents were used to treat metastatic renal cell carcinoma (*n* = 2), melanoma (*n* = 2), breast carcinoma (*n* = 1), and metastatic sarcomatoid mesothelioma (*n* = 1). Best-corrected visual acuity (BCVA) at presentation ranged from 20/25 to 20/100. Ocular diagnoses included anterior uveitis in 3 patients (1 non-granulomatous and 2 granulomatous) (Fig. 1) and granulomatous panuveitis in 3 patients.

Table 1 summarizes the demographic and clinical characteristics of the cohort.

**Table 1:** shows clinical characteristics of our cohort

Patient	Age	Gender	BCVA at presentation	Ocular event after dose/ days	ICI	Systemic diagnosis	Ocular diagnosis (OU)	Treatment and duration in weeks	Duration of follow up in months	BCVA at final visit
1	52	F	OD 20/63 OS 20/50	3rd dose, 2 days	Ip&Ni	Metastatic RCC	Non granulomatous AU	Topical steroids 6 weeks	9	OU 20/32
2	72	M	OD 20/50 OS 20/50 OD 20/40 OS 20/40	2nd dose, 14 days 4th dose, 7 days	Ip&Ni Ip&Ni	Metastatic sarcomatoid mesothelioma	Granulomatous AU	Topical steroids 8 weeks Topical steroids 6 weeks	22	OU 20/40 OU 20/40
3	55	F	OD 20/32 OS 20/25	2nd dose, 4 days	Ip&Ni	Right maxillary sinus melanoma	Granulomatous AU	Topical steroids 8 weeks	30	OD 20/20 OS 20/25
4	75	M	OD 20/32 OS 20/40	1st dose, 14 days	Pe	Metastatic RCC	Bilateral PU	Oral and topical steroids, atropine 8 weeks	72	OD 20/50 OS 20/40
5	69	F	OD 20/100 OS 20/80	10th dose, 10 days	Pe	Stage 3 C (pT4bN2b) malignant melanoma	Bilateral PU	Oral and topical steroids 8 weeks	37	OD 20/25 OS 20/32
6	65	F	OD 20/32 OS 20/32	6th dose, 7 days	Pe	cT1c-2 N0 Right Breast TNBC	Bilateral PU	Oral steroids 8 weeks	8	OD 20/20 OS 20/25

M- Male; F- female; BCVA- Best corrected visual acuity; OD- Right eye; OS- Left eye; OU- Both eyes; Ip- Ipulimumab; Ni –Nivolumab; Pe- Pembrolizumab; AU- Anterior uveitis; PU- Panuveitis; RCC- renal cell carcinoma; Topical steroids – Prednisolone acetate 1%; oral steroids- Tab prednisolone 40 mg in a tapering dose over 6 weeks

Patient 4 &5 at presentation had anterior chamber cells but not during our evaluation

*Topical steroids were prescribed 2hourly for 1st 24 h for patients 1–5 and tapered to 3 hourly the following day for a week and then tapered over next 5^-7 weeks

# Oral steroids were started at 60 mg/day and tapered over 6 weeks in panuveitis patients

Patients 4 and 5 had been offered intravenous steroids during their presentation, which had been declined by the patients

Two of the panuveitis (patient 4&5) presented to us at the stage of sunset glow fundus with their previous records showing features of panuveitis without exudative retinal detachment.

**Fig. 2:**
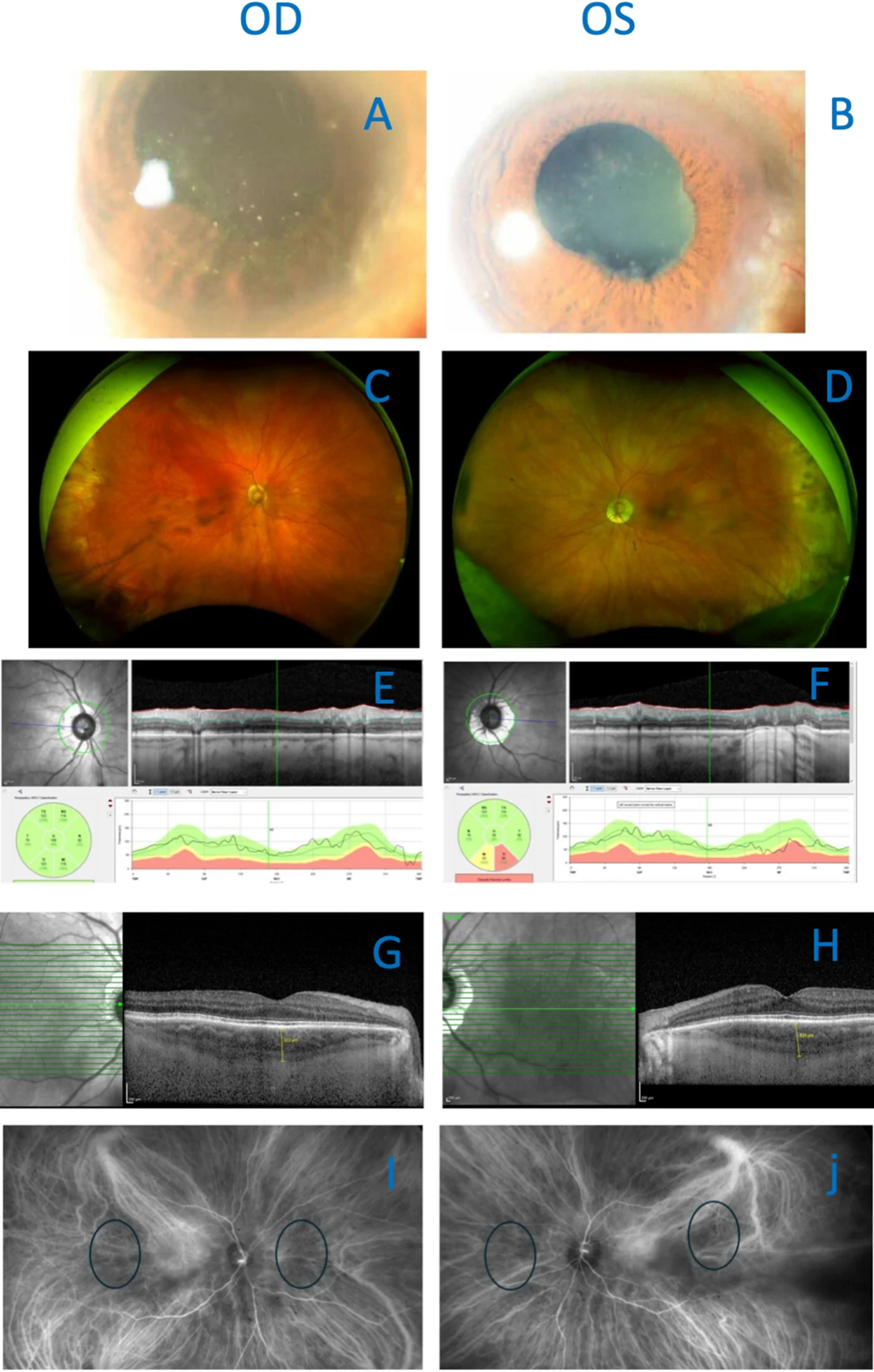
showing multimodal imaging of patient 4. **A**, **B** showing granulomatous keratic precipitates on a diffuse slit lamp imaging. **C**, **D** Widefield fundus colour photographs with sunset glow fundus and peripapillary atrophy. **E**, **F** Optical coherence tomography of optic nerve head with OS showing inferior thinning. **G**, **H** Optical coherence tomography of macula showing subfoveal choroid thickness OD 323 microns and OS 339 microns. **I**, **J** show the ICG features of choroidal granulomas represented as hypofluoroscent dark spots (black oval) at late phases

Figure 2 showing the multimodal imaging findings in patient 4.

**Fig. 3:**
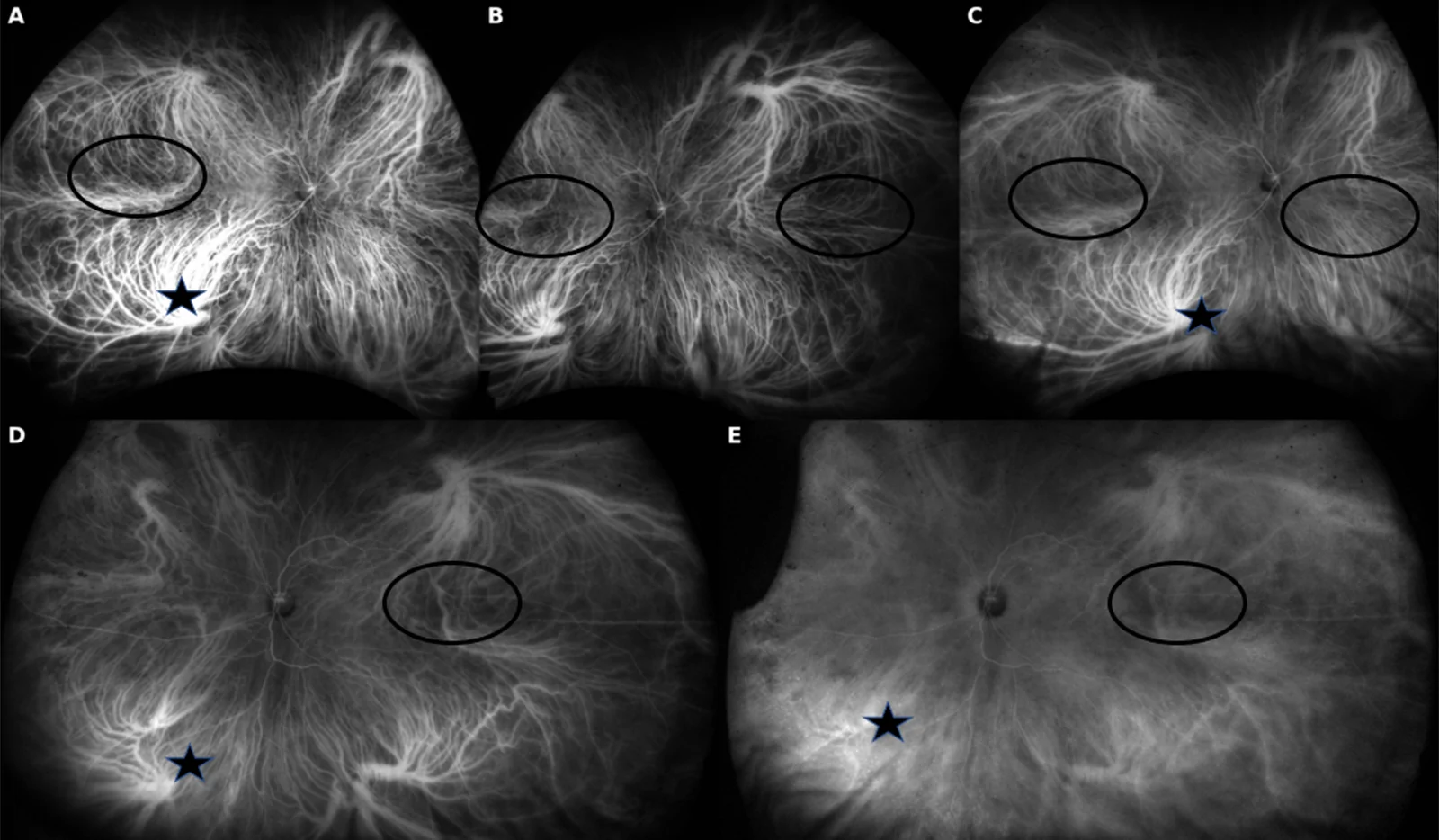
Indocyanine green angiography of patient 6 showing. Top row OD. **A**, **B**- early stromal vessel hyperfluorescence and fuzzy large choroidal vessels (black star). Persistent hypofluorescent dark dots (HDDs), (black oval) representing choroidal granulomas remain distinctly hypofluorescent, best visualized against fading background fluorescence. **C** choroidal filling delays: Incomplete perfusion with patchy hypofluorescence. Bottom row OS. **D**, **E** choroidal filling delays: Persistent hypofluorescent dark dots (HDDs), (black oval) Incomplete perfusion with patchy hypofluorescence and stromal vessel hyperfluorescence in late phases (black star), disc hyperfluorescence: Mild-moderate optic nerve staining

Optical coherence tomography showed no choroidal thickening in anterior uveitis cases. In contrast, panuveitis patients had significant choroidal thickening without exudative retinal detachment or retinal pigment epithelial undulations. Fundus fluorescein angiography revealed retinal pigment epithelium window defects in patients 4 and 5 but was normal in patient 6. Indocyanine green angiography in panuveitis cases demonstrated discrete hypofluorescent dark dots suggestive of choroidal granulomas across both posterior pole and periphery, accompanied by early stromal vessel hyperfluorescence and fuzzy large choroidal vessels. Patient 6 showed bilateral angiographic evidence of choroidal granulomas without corresponding clinical fundus abnormalities. (Fig. 3)

Table 2 shows Naranjo criteria [15] applied to our cohort.

**Table 2:** shows the Naranjo criteria applied to our cohort

Question	Yes	No	Do Not Know / Not Done	Score					
				Patient 1	Patient 2	Patient 3	Patient 4	Patient 5	Patient 6
1. Are there previous conclusive reports on this reaction?	+ 1	0	0	+ 1	+ 1	+ 1	+ 1	+ 1	+ 1
2. Did the adverse event appear after the suspected drug was administered?	+ 2	– 1	0	+ 2	+ 2	+ 2	+ 2	+ 2	+ 2
3. Did the adverse reaction improve when the drug was discontinued or a specific antagonist was administered?	+ 1	0	0	0	0	0	0	0	0
4. Did the adverse reaction reappear when the drug was re-administered?	+ 2	– 1	0	– 1	+ 2	– 1	– 1	– 1	– 1
5. Are there alternative causes (other than the drug) that could on their own have caused the reaction?	– 1	+ 2	0	+ 2	+ 2	+ 2	+ 2	+ 2	+ 2
6. Did the reaction reappear when a placebo was given?	– 1	+ 1	0	0	0	0	0	0	0
7. Was the drug detected in blood (or other fluids) in concentrations known to be toxic?	+ 1	0	0	0	0	0	0	0	0
8. Was the reaction more severe when the dose was increased, or less severe when the dose was decreased?	+ 1	0	0	0	0	0	0	0	0
9. Did the patient have a similar reaction to the same or similar drugs in any previous exposure?	+ 1	0	0	0	+ 1	0	0	0	0
10. Was the adverse event confirmed by any objective evidence?	+ 1	0	0	0	0	0	0	0	0
Total score				4	8	4	4	4	4

≥ 9 Definite ADR The reaction is almost certainly due to the medication

5 to 8 Probable ADR The reaction is likely due to the medication

1 to 4 Possible ADR The reaction could be related to the medication, but other factors are also likely causes

≤ 0 Doubtful ADR The reaction is unlikely to be related to the medication

Our cohort score 4.66

All patients were treated with topical and/or systemic corticosteroids, and none required discontinuation of ICI therapy. Patient 2 with granulomatous anterior uveitis experienced recurrence two months after the initial episode, which resolved with topical corticosteroids. No further recurrences were observed during follow-up. Patient 5 developed vitiligo on her face and back 2 years after the onset of panuveitis. No poliosis was observed.

At final follow-up of our cohort, BCVA ranged from 20/20 to 20/50, and no patient experienced significant morbidity. Follow-up duration ranged from 8 to 72 months. Ocular inflammation resolved with medical therapy in all patients, and none required long-term immunosuppression.

## Discussion

The ocular surface is the most common site of ICI related toxicity. Patients may develop keratoconjunctivitis sicca, meibomian gland dysfunction, conjunctival hyperemia and punctate epithelial erosions, sometimes in association with systemic sicca features [16, 17]. Uveitis is one of the most common ocular irAEs associated with ICI—one study calculated uveitis made up almost 70% of all ocular irAEs, of which more than 80% were bilateral at diagnosis [18]. In a retrospective cohort study of the Medicare database, patients receiving ICI therapy for cancer were two times more likely to develop non-infectious uveitis than their non-ICI counterparts [19]. The Common Terminology Criteria for Adverse Events (CTCAE) is used to grade uveitis resulting from irAEs of cancer treatments [20]. 

Table 3 shows Common Terminology Criteria for Adverse Events (CTCAE) Version 4.0 the grading of uveitis [20]. Our cohort had 3 patients with anterior uveitis with grade 2 and 3 patients with panuveitis with grade 3 criteria. Four types of uveitis are found in association with ICIs namely anterior, intermediate, posterior and panuveitis [21] which is similar to the working group description of uveitis [22]. 

**Table 3:** Grading of Uveitis According to the CTCAE Criteria [20].

Grade 1	Grade 2	Grade 3	Grade 4
Anterior uveitis with trace cells	Anterior uveitis with 1 + or 2 + cells	Anterior uveitis with 3 + or greater cells; intermediate posterior or pan-uveitis	Best corrected visual acuity of 20/200 or worse in the affected eye

In a literature review of 241 eyes that were diagnosed with uveitis following ICI therapy, anterior uveitis was the most common type of uveitis (37.7%), followed by panuveitis (34.0%), posterior uveitis (25.7%), and finally intermediate uveitis (1%). This distribution was similar regardless of the ICI used, but patients under a combination of ICIs demonstrated a higher rate of anterior uveitis [23]. Martens et al. [24]. in their review of 290 published ocular adverse events found uveitis to be the most frequently reported manifestation, accounting for 46.2% of cases, with melanoma representing the underlying malignancy in nearly two-thirds of patients and nivolumab and ipilimumab being the agents most commonly implicated. They noted a strong discrepancy between anterior and panuveitis, and intermediate uveitis. Among 134 reported cases, they found ICI-induced anterior uveitis accounted for 45%, panuveitis 42% (of which 22% were VKH-like), posterior 4%, and intermediate 6% [24]. More recent population-level evidence confirmed that melanoma carries a two to three times more likely risk of ICI-associated non-infectious uveitis than other malignancies, suggesting that both tumour biology and drug-related immune activation contribute to disease development [25, 26]. Lung cancer and renal cell carcinoma are also associated with uveitis is, but its risk is much lower than melanoma [11]. 

Clinically, the spectrum ranges from isolated anterior uveitis to severe bilateral panuveitis and VKH-like syndromes, often presenting within the first few months of treatment. Akcay et al. [27]. described a patient who developed bilateral granulomatous panuveitis with choroidal inflammation after combined nivolumab and ipilimumab therapy, requiring high-dose systemic corticosteroids and discontinuation of immunotherapy [27]. Similarly, Diamantopoulos et al. [28]. reported an elderly woman who developed incomplete VKH disease with bilateral serous retinal detachments and neurosensory hearing loss following encorafenib and binimetinib therapy for metastatic melanoma, illustrating how melanocyte-targeted immune responses can extend beyond classical ICIs to targeted therapies used in melanoma management [28]. Collectively, these studies suggest that ocular inflammation in melanoma may reflect shared antigens between melanoma cells and melanocytes within the uvea, creating a predisposition to autoimmune ocular toxicity. Early recognition, prompt ophthalmic assessment, and close collaboration between ophthalmologists and oncologists are therefore essential to preserve vision while balancing the need for ongoing cancer control [24–28]. Various studies analyzed the contribution of ICI class on the risk of drug-related uveitis. A study analyzing the FDA Adverse Events Reporting System (FAERS) found ipilimumab and nivolumab combination therapy to have the strongest association with uveitis, along with others which showed CTLA-4 inhibitors were associated with higher risks of anterior uveitis [2]. Kim et al. [29] in their study of 40 ICI-induced uveitis cases found pembrolizumab therapy was more highly associated with intraocular inflammation, although the analysis did not differentiate between anterior and posterior uveitis and included ophthalmoplegia. PD-1/PD-L1 inhibitors seem to be differentially associated with posterior uveitis [29]. 

The severity of visual acuity disturbance can vary in ICI induced uveitis, ranging from 20/20 vision to light perception, but the median visual acuity tends to be around 20/40 [23]. 

Duma et al. [30] Women with metastatic melanoma and non-small cell lung carcinoma are more likely to experience irAEs compared with men.

Vogt–Koyanagi–Harada syndrome is a rare form of bilateral panuveitis that occurs when inflammation is triggered against melanocyte-containing choroidal tissue and spreads into neighbouring structures [31]. It is characterized by granulomatous anterior or posterior uveitis, with thickening of the choroid, papillitis, and serous retinal detachment, possibly associated with central nervous system, auditory, or dermatological manifestations. Depigmentation of the fundus and limbus several weeks after the episode may leave characteristic sunset-like lesions [1]. Zhang et al. [2] in their review analyzing VKH-like uveitis in the context of ICI therapy, totalling 41 patients found most were women with a mean age of 68 years old, being treated for melanoma (59%) with a PD-1 or PD-L1 inhibitor (76%). There is a high incidence of HLA-DR4 or HLA-DRB1 positivity (37%), and the actual value may be higher than reported [2]. 

The human leukocyte antigen (HLA) gene, encoded on chromosome 6 has many variants in the human population, and some have been preferentially associated with autoimmune diseases [32–35]. The allele is strongly associated with autoimmune diseases and 35% of VKH patients in the general population, with one study finding 100% specificity for VKH in a study of 63 Japanese patients [36–38]. In our cohort HLA typing was not done.

### Clinical phenotypes

Granulomatous ocular inflammation observed during ICI therapy may represent either a VKH-like syndrome or a sarcoid-like reaction (SLR), two entities that share overlapping clinical features but differ in their underlying immunopathogenesis and systemic association. Chaudot et al. [18] reported a series characterized by predominantly granulomatous features, which is mirrored in our cohort (5 out of 6 patients). This is a distinct observation, as other larger pharmacovigilance studies have more frequently reported non-granulomatous phenotypes [39, 40]. VKH-like disease is thought to result from T-cell-mediated autoimmune responses directed against melanocyte-associated antigens, leading to diffuse choroidal inflammation, sunset-glow fundus, vitiligo, poliosis and, occasionally, neurological or auditory manifestations [2, 11, 31]. 

In contrast, SLR is characterized by the formation of non-caseating granulomas resulting from dysregulated Th1-mediated immune activation and closely resembles systemic sarcoidosis [39–41]. Patients with SLR commonly develop mediastinal or hilar lymphadenopathy, pulmonary infiltrates, elevated serum angiotensin-converting enzyme levels, and biopsy-proven non-caseating granulomas, while ocular manifestations may include granulomatous anterior uveitis, retinal vasculitis, choroidal granulomas and panuveitis [42–45]. They are most often seen within a few months of starting treatment, may involve the lungs, lymph nodes, and skin, and can occur across different cancer types rather than only melanoma. Most cases are mild and either improve on their own or respond to corticosteroids, and immunotherapy often can be continued [42–45]. The practical challenge is to recognize these lesions early so they are not mistaken for cancer progression, which could lead to unnecessary stopping of an effective immunotherapy [44, 45]. 

None of our patients demonstrated hilar lymphadenopathy on standard chest X-rays; however, this imaging technique offers only moderate sensitivity for detecting such findings relative to a chest computed tomography scan. When evaluating what actually caused the inflammation in our patients, we had to carefully weigh the role of their underlying cancers. This was especially true for our two patients with metastatic melanoma. Melanoma can independently trigger an autoimmune response against melanocytes, leading to melanoma-associated retinopathy or VKH-like inflammation even without immunotherapy. This likely reflects shared melanocyte-related antigens, such as MART-1/Melan-A, which can be recognized by immune cells in both melanoma and the retina [46]. Melanoma-associated retinopathy (MAR) is a rare paraneoplastic autoimmune manifestation of cutaneous malignant melanoma. Patients classically present with acute onset night blindness, positive visual phenomena and visual field defects, and typically have significantly reduced quality of life as a result [47, 48]. Our patients did not show these features. MAR may precede or follow the diagnosis of melanoma. Patients with MAR have the same antigenic retinal proteins that have been associated with cancer-associated retinopathy [46]. 

The clinical picture in our cohort points firmly to the drugs themselves. For one, the timeline was striking: in every case, the uveitis flared up only after the patients started their immunotherapy, consistently appearing within a tight, biologically plausible window of 2 to 14 days. This sudden onset wasn’t just based on patient symptoms; it was backed by hard, objective evidence on multimodal imaging, such as active choroidal granulomas seen on indocyanine green angiography and marked choroidal thickening on OCT.

Perhaps the strongest argument for drug toxicity is that we saw these severe granulomatous reactions across a wide variety of cancers—not just melanoma, but also renal cell carcinoma, breast carcinoma, and mesothelioma. We also observed it across different classes of ICIs, including both pembrolizumab and combination ipilimumab-nivolumab therapy.

While we can never entirely rule out a baseline, tumor-driven autoimmune response in the melanoma patients, the overarching clinical narrative is hard to ignore. The strict timing of the drug administration, the objective imaging findings, and the fact that identical inflammatory patterns appeared in completely different cancers all give us high confidence that these ocular events were directly driven by the ICIs.

### Multimodal imaging and VKH-like features

Using multimodal imaging gave us critical diagnostic insights into our cohort. As the use of ICIs continues to expand in Asia, ophthalmologists should maintain a high index of suspicion for VKH-like disease, particularly in patients presenting with granulomatous inflammation or subtle choroidal findings [13]. 

One notable finding was the appearance of a “sunset-glow fundus” in two patients with panuveitis, which is a classic hallmark of VKH-like disease [13]. Although many ICI-induced VKH-like cases in the literature fail to meet strict diagnostic criteria because of incomplete documentation [13], our cohort demonstrated definitive angiographic evidence of the disease. Subfoveal choroidal thickness (SFCT) closely reflects inflammatory activity in VKH, with marked choroidal thickening during the acute phase and progressive thinning following successful treatment [49, 50]. Moreover, swept-source OCT has emerged as a useful non-invasive tool for monitoring disease activity, enabling detection of subclinical recurrences. Increased choroidal thickness has been associated with posterior segment relapse, whereas choroidal thinning correlates with treatment response and disease quiescence [51]. 

In our series, choroidal involvement was confined exclusively to patients with panuveitis; none of the anterior uveitis cases showed choroidal thickening on OCT. Interestingly, both panuveitis patients had markedly thickened choroids despite being at the sunset glow stage and lacking any signs of exudative retinal detachment or retinal pigment epithelial undulations. This stands out because a sunset glow fundus is typically viewed as a manifestation of chronic disease, yet our findings show that substantial choroidal inflammation can linger even without obvious exudative changes.

This is where ICGA proved especially valuable and revealed hypodense dark dots—representing early, melanocyte-rich choroidal granulomas—in all three of our panuveitis patients. Strikingly, in one case, these angiographic findings appeared before there were any visible clinical abnormalities on the fundus. This really highlights just how sensitive ICGA is as a tool for catching subclinical choroidal involvement early. Furthermore, when patient 5 later developed cutaneous hypopigmentation, it added weight to the theory that T-cell cross-reactivity is actively targeting melanocytic tissues in both the eyes and the skin [45, 52]. Ultimately, seeing persistent choroidal thickening alongside these granulomas tells us that choroidal inflammation is still ongoing, even if the fundus appears clinically quiet and convalescent. This is a crucial reminder: just because a sunset glow fundus looks stable, we shouldn’t assume the immune system has reached complete quiescence. Instead, combining OCT with ICGA gives us a much more accurate picture of residual choroidal activity, which can better guide our treatment decisions. Since chronic VKH is generally tied to progressive choroidal thinning and lower SFCT values compared to healthy eyes [53, 54]. the persistent choroidal thickening we observed in our panuveitis patients is a highly noteworthy anomaly. American Society of Clinical Oncology (ASCO) recommendations for specific organ system-based toxicity diagnosis and management are available [55–57]. However, it does not have a separate ocular grading system. Ocular irAEs are graded using CTCAE criteria [20], and the ASCO irAEs guideline then uses those grades to determine management. While management varies according to the organ system affected, in general, ICI therapy should be continued with close monitoring for grade 1 toxicities, except for some neurologic, hematologic, and cardiac toxicities. ICI therapy may be suspended for most grade 2 toxicities, with consideration of resuming when symptoms revert ≤ grade 1. Corticosteroids may be administered. Grade 3 toxicities generally warrant suspension of ICI is and the initiation of high-dose corticosteroids. Corticosteroids should be tapered over the course of at least 4–6 weeks. Some refractory cases may require other immunosuppressive therapy. In general, permanent discontinuation of ICI is recommended with grade 4 toxicities, except for endocrinopathies that have been controlled by hormone replacement. With a retrospective data set, we were unable to get the exact ASCO grading for other systemic irAEs. In consultation with the treating oncologist, oral steroid therapy was given for patients with panuveitis. However, the outcomes in our cohort suggest a more optimistic management pathway with grade 2 in anterior uveitis patients and grade 3 in panuveitis patients [20, 55–57]. Despite the severity of presentation in our panuveitis cases (BCVA as low as 20/100), all six patients responded well to topical or oral systemic corticosteroids.

Crucially, none of our patients required the discontinuation of their ICI therapy. This aligns with findings from the Mayo Clinic cohort, which indicated that most ocular irAEs can be successfully managed through close collaboration between ophthalmology and oncology teams [9]. Our results demonstrate that with prompt recognition and aggressive medical management, visual function can be preserved (final BCVA 20/20 to 20/50) without compromising the patient’s systemic oncological treatment.

### Limitations

Our study has a few important limitations. First, it is a small, retrospective review of just six patients treated at a single centre. As our cohort included a heterogeneous mix of malignancies and ICI regimens, and lacked a control group, it is difficult to draw broad generalizations about the effects of specific drugs. Furthermore, since all our patients were Chinese Singaporeans, the strong tendency toward granulomatous and VKH-like features we observed might not fully represent other ethnic groups.

The retrospective design also meant we had to deal with incomplete clinical data; for instance, formal American Society of Clinical Oncology (ASCO) systemic irAEs grading and HLA typing were unavailable in the records. We also have to consider referral bias, as patients with milder or self-limiting symptoms likely never made it to our specialist clinic. Our ocular imaging approach was not uniform, either. Because we primarily reserved fluorescein and indocyanine green angiography for patients already showing panuveitis, we may have missed subclinical choroidal involvement in other cases though OCT did not show choroidal thickening in the anterior uveitis patients.

Finally, while none of our patients showed radiographic evidence of systemic sarcoidosis on a standard chest X-ray, an ICI-associated sarcoid-like reaction remains a crucial differential diagnosis for these granulomatous presentations. Unfortunately, the retrospective nature of our series precluded us from pursuing more definitive workups, such as a chest CT or tissue biopsy.

Despite these drawbacks, we believe our detailed clinical characterization and long-term follow-up still offer a valuable look at how ICI-associated ocular inflammation presents in an Asian population, particularly by highlighting just how useful ICGA can be for catching hidden choroidal disease.

## Conclusion

In this Singapore cohort, we document a distinct pattern of ICI-induced ocular inflammation that is predominantly granulomatous, presenting primarily as anterior uveitis or panuveitis. Multimodal imaging proved absolutely essential for these patients. By utilizing ICGA, we were able to unmask choroidal granulomas and closely track the evolution of sunset-glow fundi in those with panuveitis. Fortunately, the clinical outcomes were highly encouraging. Using only topical or systemic corticosteroids, all six patients achieved favorable final visual acuities ranging from 20/20 to 20/50. Most importantly, over a follow-up period of 8 to 72 months, we successfully managed these severe inflammatory events without ever needing to interrupt their life-saving ICI therapy. Ultimately, these findings highlight just how crucial early, multidisciplinary management is, especially when treating diverse Asian cohorts. By early referral and promptly managing, we can preserve our patients’ vision without compromising their oncologic care. It is a dual success that strongly supports refining current clinical guidelines, allowing for the safe continuation of immunotherapy even while managing severe uveitis.

## Data Availability

No datasets were generated or analysed during the current study.
